# *Wilms Tumor 1* Mutations Are Independent Poor Prognostic Factors in Pediatric Acute Myeloid Leukemia

**DOI:** 10.3389/fonc.2021.632094

**Published:** 2021-04-21

**Authors:** Yin Wang, Wen-Jun Weng, Dun-Hua Zhou, Jian-Pei Fang, Srishti Mishra, Li Chai, Lu-Hong Xu

**Affiliations:** ^1^ Department of Pediatrics, Sun Yat-sen Memorial Hospital, Sun Yat-sen University, Guangzhou, China; ^2^ Guangdong Provincial Key Laboratory of Malignant Tumor Epigenetics and Gene Regulation, Sun Yat-sen Memorial Hospital, Sun Yat-sen University, Guangzhou, China; ^3^ Cancer Science Institute, Yong Loo Lin School of Medicine, National University of Singapore, Singapore, Singapore; ^4^ Department of Pathology, Brigham & Women’s Hospital, Harvard Medical School, Boston, MA, United States

**Keywords:** acute myeloid leukemia, *WT1* mutations, pediatric patients, prognostic factors, *FLT3*/ITD mutations

## Abstract

The prognostic impact of *Wilms tumor 1* (*WT1*) mutations remains controversial for patients with acute myeloid leukemia (AML). Here, we aimed to determine the clinical implication of *WT1* mutations in a large cohort of pediatric AML. The clinical data of 870 pediatric patients with AML were downloaded from the therapeutically applicable research to generate effective treatment (TARGET) dataset. We analyzed the prevalence, clinical profile, and prognosis of AML patients with *WT1* mutations in this cohort. Our results showed that 6.7% of total patients harbored *WT1* mutations. These *WT1* mutations were closely associated with normal cytogenetics (*P*<0.001), FMS-like tyrosine kinase 3/internal tandem duplication (*FLT3*/ITD) mutations (*P*<0.001), and low complete remission induction rates (*P*<0.01). Compared to the patients without *WT1* mutations, patients with *WT1* mutations had a worse 5-year event-free survival (21.7 ± 5.5% vs 48.9 ± 1.8%, *P*<0.001) and a worse overall survival (41.4 ± 6.6% vs 64.3 ± 1.7%, *P*<0.001). Moreover, patients with both *WT1* and *FLT3*/ITD mutations had a dismal prognosis. Compared to chemotherapy alone, hematopoietic stem cell transplantation tended to improve the prognoses of *WT1*-mutated patients. Multivariate analysis demonstrated that *WT1* mutations conferred an independent adverse impact on event-free survival (hazard ratio 1.910, *P* = 0.001) and overall survival (hazard ratio 1.709, *P* = 0.020). In conclusion, our findings have demonstrated that *WT1* mutations are independent poor prognostic factors in pediatric AML.

## Introduction

Acute myeloid leukemia (AML) is a type of blood cancer that originates in the bone marrow from immature white blood cells known as myeloblasts. About 20% of all children with leukemia have AML (1, 2). In the last few years, collaborative studies have revealed a link between the degree of genetic heterogeneity of AML and the clinical outcome, allowing risk stratification before therapy and guiding post-induction treatment (3). The *Wilms tumor 1* (*WT1*) gene, located on chromosome 11p13, encodes a zinc-finger protein that exists in multiple isoforms. It has been implicated in the regulation of cell survival, proliferation and differentiation, and may function both as a tumor suppressor and an oncogene (4, 5). Various mutations across *WT1* gene have been reported in solid tumors and AML (6, 7). However, the prognostic impact of *WT1* mutations remains controversial for patients with AML (8).

The *WT1* mutations have been shown to be independent predictors of worse clinical outcome in some but not all adult AML studies (9–11). Recently, *WT1* mutations are proposed to be prognostic markers of risk stratification for adult AML (12). However, the prognostic implications of *WT1* mutations have not been clarified in pediatric AML. Moreover, large cohort studies on the clinical significance of *WT1* mutations in pediatric AML are scarce. A pediatric study of 298 patients with AML found that *WT1* mutations conferred an independent poor prognostic significance (13). However, another study of 842 pediatric AML revealed that the presence of *WT1* mutations had no independent prognostic significance in predicting the disease outcome (14). Recently, in a cohort of 353 pediatric patients with AML, Niktoreh et al. (15) have found that *WT1* mutations significantly increased the chance of relapse or treatment failure and reduced the probability of 3-year overall survival (OS), but had no significant impact on the 3-year probability of event-free survival (EFS). On the other hand, hematopoietic stem cell transplantation (HSCT) is an important treatment modality for patients with AML. However, the role of HSCT for patients with *WT1* mutations remains unknown.

To determine the clinical implication of *WT1* mutations, an independent large cohort study of pediatric AML is needed. Therefore, we analyzed the clinical data of 870 pediatric patients with AML from the therapeutically applicable research to generate effective treatment (TARGET) dataset. We found that *WT1* mutations are independent poor prognostic factors in pediatric AML in terms of 5-year EFS and OS. Patients with both *WT1* and FMS-like tyrosine kinase 3/internal tandem duplication (*FLT3*/ITD) mutations had a dismal prognosis. Moreover, HSCT might be an effective strategy for patients with *WT1* mutations.

## Materials and Methods

### Patients

The clinical data on patients with AML were downloaded from the TARGET dataset (https://ocg.cancer.gov/programs/target/data-matrix). In total, 870 pediatric patients younger than 18 years old with the information of *WT1* mutations were included in our study. The year of diagnosis ranged from 1996 to 2010 while the year of last follow-up ranged from 1997 to 2015. The diagnosis of pediatric AML and risk stratification were defined according to the Children’s Oncology Group (COG) guidelines. Subtype classifications of AML were assigned according to the French–American–British (FAB) classifications. Mutation analyses of *WT1*, *FLT3*/ITD, *NPM1*, and *CEBPA* were performed as previously described (14, 16–18). Treatment protocols for AML included AAML03P1, AAML0531 and CCG-2961. HSCT was considered for high-risk patients in the first complete remission. Detailed treatments and risk stratification of these studies have been previously described (19).

### Statistical Analysis

The data were analyzed with the Statistical Package for the Social Sciences (SPSS^®^) version, 20.0 (IBM Corporation, Armonk, NY, USA). The χ2 test was used to compare the frequencies of mutations. Fischer’s exact test was used when data were sparse. The nonparametric Mann–Whitney *U*-test was applied for continuous variables. Complete remission (CR) was defined as bone marrow aspirate with < 5% blasts by morphology. EFS was defined as the time between diagnosis and first event, including induction failure, relapse, or death of any cause. OS was defined as the time between diagnosis and death from any cause. The survival curves were estimated using the Kaplan–Meier method and compared using the log-rank test. Cox proportional hazard models were used to estimate hazard ratios (HR) for multivariate analyses. A two-sided *P*-value less than 0.05 was considered statistically significant for all statistical analyses.

## Results

### Relationship Between *WT1* Mutations and Clinical Characteristics

The patients’ clinical characteristics are shown in **Table 1**. Overall, among the 870 pediatric patients with AML, 58 patients (6.7%) were identified with *WT1* mutations. The white blood cell count (WBC) at diagnosis was significantly higher in *WT1*-mutated patients (median 56.9×10^9^/L) than in *WT1* wild-type patients (median 30.8×10^9^/L; *P*=0.041). In *WT1*-mutated group, the FAB subtypes were mainly M1, M2, and M4. A higher proportion of *WT1*-mutated patients had M4 morphology in comparison with *WT1* wild-type patients (41.2% vs 25.9%; *P* = 0.018). We also evaluated the associations between *WT1* mutations and cytogenetic and molecular alterations. In terms of cytogenetics, *WT1* mutations were found more frequently in the normal cytogenetics subset (44.2% of *WT1*-mutated patients had normal cytogenetics compared with 22.3% of those without *WT1* mutations; *P*<0.001). Regarding the molecular alterations, there was a substantial overlap between *WT1* mutations and *FLT3*/ITD, as shown in **Table 1**, 48.3% of those carrying a *WT1* mutation were also *FLT3*/ITD positive as opposed to 14.7% of patients without *WT1* mutations (*P*<0.001). Moreover, the *WT1-*mutated patients were classified more frequently as high risk (40.7% vs 12.6%; *P*<0.001). The treatment protocols for pediatric AML were equally distributed between these two groups (*P*=0.058). However, there were no significant differences in the median age, the median of *FLT3*/ITD allelic ratio, *NPM1*, and *CEBPA* mutations between the *WT1*-mutated group and *WT1* wild-type group.

**Table 1:** Characteristics of pediatric patients with or without *WT1* mutations.

	All patients	*WT1*-mutated case	*WT1* wildtype case	*P*-value
Number (%)	870	58 (6.7%)	812(93.3%)	
Age, median (year)	9.6	11	9.5	0.221
<3years, n (%)	211(24.3%)	6 (10.3%)	205 (25.2%)	0.011
3≤Age<10years, n (%)	237(27.2%)	19 (32.8%)	218 (26.8%)	0.329
10≤Age<18years, n (%)	422(48.5%)	33 (56.9%)	389 (47.9%)	0.186
Sex				0.119
male, n (%)	454 (52.2%)	36 (62.1%)	418 (51.5%)	
female, n (%)	416 (47.8%)	22 (37.9%)	394 (48.5%)	
WBC, ×10^9^/L,				
Median (range)	31.7(0.2-610)	56.9(1.1-446)	30.8(0.2-610)	0.041
FAB classification: n (%)				0.001
M0	20 (2.8%)	1 (2.0%)	19 (2.9%)	>0.999
M1	96 (13.4%)	10 (19.6%)	86 (13.0%)	0.181
M2	193 (27.0%)	11 (21.6%)	182 (27.5%)	0.362
M3	2 (0.3%)	0 (0.0%)	2 (0.3%)	>0.999
M4	193 (27.0%)	21 (41.2%)	172 (25.9%)	0.018
M5	160 (22.4%)	3 (5.9%)	157 (23.7%)	0.003
M6	11 (1.5%)	4 (7.8%)	7 (1.1%)	0.005
M7	39 (5.5%)	1 (2.0%)	38 (5.7%)	0.351
Risk group: n (%)				<0.001
Low risk	328 (39.0%)	15 (27.8%)	313 (39.8%)	0.079
Standard risk	391 (46.5%)	17 (31.5%)	374 (47.6%)	0.022
High risk	121 (14.4%)	22 (40.7%)	99 (12.6%)	<0.001
*FLT3*/ITD				<0.001
Positive, n (%)	147 (16.9%)	28 (48.3%)	119 (14.7%)	
Negative, n (%)	722(83.1%)	30 (51.7%)	692 (85.3%)	
*FLT3*/ITD allelic ratio, Median (range)	0.54	0.55	0.54	0.865
	(0.03-9.50)	(0.03-5.19)	(0.03-9.50)	
*NPM1*				0.794
Positive, n (%)	66(7.6%)	3(5.3%)	63(7.8%)	
Negative, n (%)	802(92.4%)	63(94.7%)	748(92.2%)	
*CEBPA*				0.245
Positive, n (%)	49(5.7%)	1(1.7%)	48(5.9%)	
Negative, n (%)	817(94.3%)	57(98.3)	760(94.1%)	
Cytogenetic status				
Normal (n, %)	196(23.7%)	23(44.2%)	173(22.3%)	<0.001
Abnormal (n, %)	631 (76.4%)	29 (55.8%)	602 (77.7%)	0.317
inv(16)(n, %)	106(12.8%)	9(17.3%)	97(12.5%)	0.046
t(8;21) (n, %)	128(15.5%)	3(5.8%)	125(16.1%)	
HSCT in 1st CR				0.906
No (n, %)	663 (83.8%)	38 (84.4%)	625 (83.8%)	
Yes (n, %)	128 (16.2%)	7 (15.6%)	121 (16.2%)	
Protocol				0.058
AAML03P1 (n, %)	91 (10.5%)	7 (12.1%)	84 (10.3%)	0.679
AAML0531 (n, %)	732 (84.1%)	44 (75.9%)	688 (84.7%)	0.074
CCG-2961 (n, %)	47(5.4%)	7 (12.1%)	40 (4.9%)	0.031
CR status at end of course 1				0.002
CR, n (%)	656 (76.3%)	35 (60.3%)	621 (77.4%)	0.003
Not CR, n (%)	189 (22.0%)	20 (34.5%)	169 (21.1%)	0.017
Death, n (%)	15 (1.7%)	3 (5.2%)	12 (1.5%)	0.074
CR status at end of course 2				<0.001
CR, n (%)	736 (87.2%)	38 (69.1%)	698 (88.5%)	<0.001
Not CR, n (%)	88 (10.4%)	14 (25.5%)	74 (9.4%)	<0.001
Death, n (%)	20 (2.4%)	3 (5.5%)	17 (2.2%)	0.136

CEBPA CCAAT, enhancer binding protein alpha; CR, complete remission; FAB, French–American–British morphology classification; FLT3/ITD, internal tandem duplication of the FLT3 gene; HSCT, hematopoietic stem cell transplantation; NPM1, Nucleophosmin; WBC, white blood cell count.

### Clinical Outcome and Prognostic Effect of *WT1* Mutations

The CR rate was determined for all patients after the first and second course of induction therapy. At the end of the first course of therapy, patients with *WT1* mutations had a lower rate of CR (60.3%) compared to those without *WT1* mutations (77.4%), and the difference was statistically significant (*P*=0.002). At the end of the second course of therapy, 38(69.1%) of the 55 patients with *WT1* mutations achieved a CR compared to 698 (88.5%) of 789 patients without *WT1* mutations (*P*<0.001). Taken together, *WT1* mutations were significantly associated with low induction CR rates.

**Figure 1:**
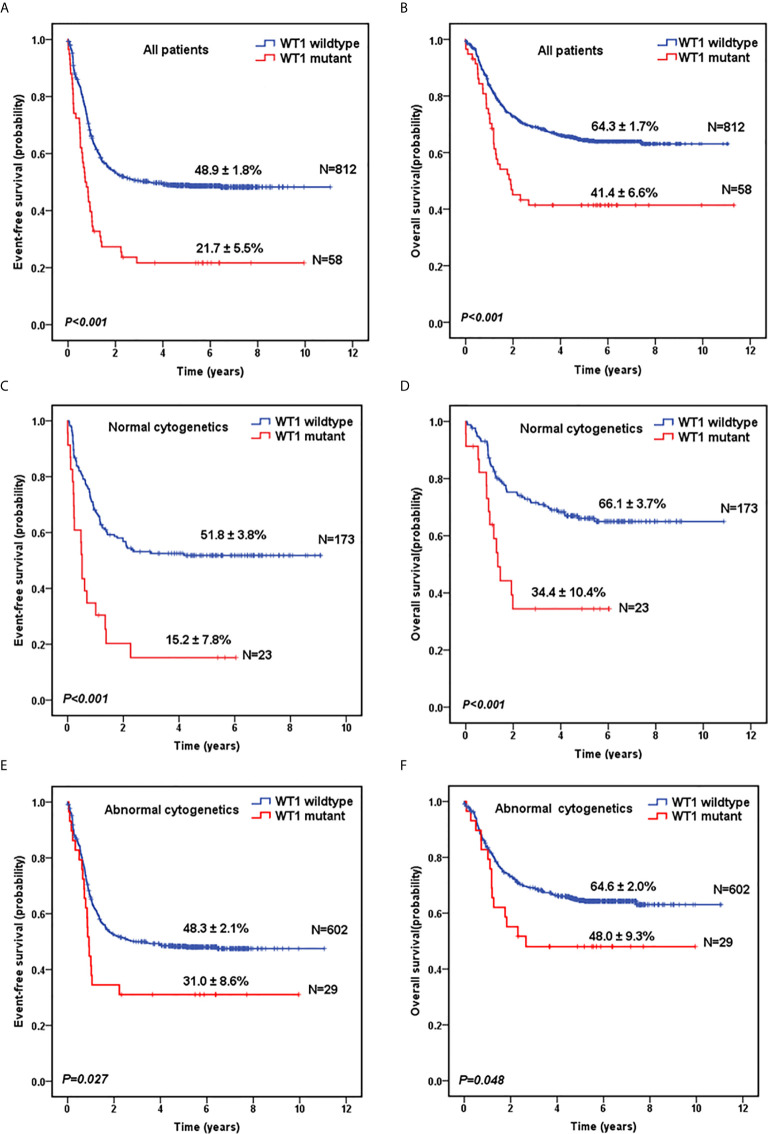
Survival curves of pediatric AML patients with and without *WT1* mutations. Probability of EFS **(A)** and OS **(B)** for all patients with and without *WT1* mutations, respectively. Probability of EFS **(C)** and OS **(D)** for cytogenetically normal patients with and without *WT1* mutations, respectively. Probability of EFS **(E)** and OS **(F)** for cytogenetically abnormal patients with and without *WT1* mutations, respectively.

Next, we evaluated the survival data for all the 870 pediatric patients. The median follow-up time for the survivors was 5.6 years. As shown in **Figure 1A**, *WT1*-mutated patients had a significantly worse 5-year EFS (21.7 ± 5.5%) compared with *WT1* wild-type patients (48.9 ± 1.8%; *P*<0.001). Moreover, patients with *WT1* mutations had a worse 5-year OS (41.4 ± 6.6%) than those without *WT1* mutations (64.3 ± 1.7%; *P*<0.001) (**Figure 1B**). When analyses were restricted to patients having normal cytogenetics, there were significant differences in the outcome between patients with and without *WT1* mutations (**Figures 1C, D**) (5-year EFS: 15.2 ± 7.8% vs 51.8 ± 3.8%, *P*<0.001; 5-year OS: 34.4 ± 10.4% vs 66.1± 3.7%, *P*<0.001). In the subgroup of abnormal cytogenetics (**Figures 1E, F**), *WT1*-mutated patients also had a worse survival time compared with *WT1* wild-type patients in terms of 5-year EFS (31.0 ± 8.6% vs 48.3 ± 2.1%, *P*=0.027) and OS (48.0 ± 9.3% vs 64.6± 2.0%, *P*=0.048).

### Prognostic Impact of *WT1* and *FLT3*/ITD Mutations

Survival data for patients with *FLT3*/ITD positive and negative were also explored. As shown in **Figure S1A**, *FLT3*/ITD positive was significantly associated with inferior EFS (5-year EFS=33.5± 4.0% vs 49.7± 1.9% for *FLT3*/ITD-negative; *P*<0.001). Moreover, the *FLT3*/ITD positive group had a worse 5-year OS (51.5 ± 4.3%) than the *FLT3*/ITD-negative group (65.0 ± 1.8%; *P*=0.003) (**Figure S1B**).

**Figure 2:**
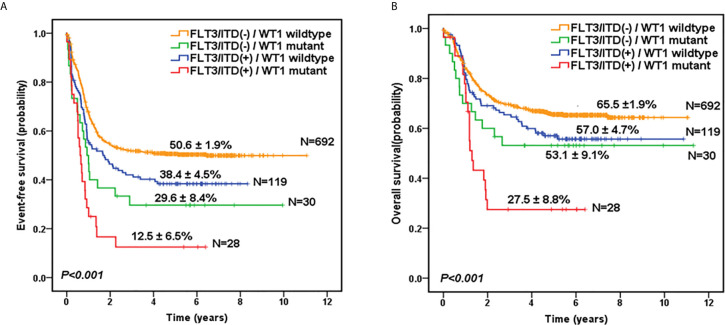
Survival curves of all pediatric AML patients according to the combined *WT1* mutations and positive FLT3/ITD status. Probability of EFS **(A)** and OS **(B)** for patients according to the combined *WT1* mutations and positive *FLT3*/ITD status, respectively.

Given the overlap between *WT1* mutations and positive *FLT3*/ITD status, subset analysis was performed to assess the relative influence of *WT1* mutations and *FLT3*/ITD on the prognosis of children with AML (**Figures 2A, B**; **Table 2**). In the *FLT3*/ITD-positive subgroup, *WT1*-mutated patients had an extremely dismal prognosis (5-year EFS =12.5 ± 6.5% vs 38.4± 4.5% for *WT1* wild-type patients, HR: 2.179 [1.364-3.482], *P*=0.001; 5-year OS = 27.5± 8.8% vs 57.0 ± 4.7% for *WT*1 wild-type patients, HR: 2.225[1.305-3.796], *P*=0.003). When restricted to the *FLT3*/ITD-negative subgroup, *WT1* mutations had an adverse impact on 5-year EFS (HR: 1.861[1.197-2.892], *P*=0.006) instead of 5-year OS (HR: 1.600[0.933-2.744], *P*=0.088). Similarly, for the *WT1* wild-type patients, *FLT3*/ITD positive had reduced 5-year EFS (HR: 1.386[1.075-1.788], *P*=0.012) but not 5-year OS (HR: 1.305[0.961-1.771], *P*=0.088). However, *FLT3*/ITD mutations had no significantly negative influence on the outcome of *WT1*-mutated patients (EFS HR: 1.605[0.886-2.906], *P*=0.118; OS HR: 1.748[0.870-3.514], *P*=0.117).

**Table 2:** Statistical comparison of survival data according to both *WT1* and *FLT3*/ITD status.

Comparison	EFS hazard ratio (95% CI)	EFS	OS hazard ratio	OS
		*P*-value	(95% CI)	*P*-value
*FLT3*/ITD(-): *WT1* wildtype vs *WT1* mutant	1.861(1.197-2.892)	0.006	1.600(0.933-2.744)	0.088
FLT3/ITD(+): *WT1* wildtype vs *WT1* mutant	2.179(1.364-3.482)	0.001	2.225(1.305-3.796)	0.003
*WT1* wildtype: *FLT3*/ITD(-)vs *FLT3*/ITD(+)	1.386(1.075-1.788)	0.012	1.305(0.961-1.771)	0.088
*WT1* mutant: *FLT3*/ITD(-) vs *FLT3*/ITD(+)	1.605(0.886-2.906)	0.118	1.748(0.870-3.514)	0.117

CI, confidence interval; EFS, event-free survival; FLT3/ITD, internal tandem duplication of the FLT3 gene; OS, overall survival.

Similar results were found in the subgroup of cytogenetically normal AML patients according to the combined *WT1* mutations and positive *FLT3*/ITD status (**Figure S2**). Of note, the survival curves showed that there were no significant differences between *WT1*-mutated patients with *FLT3*/ITD-positive (n=17) and *FLT3*/ITD negative (n=6), in terms of 5-year EFS (14.1 ± 9.0% vs 16.7 ± 15.2%; *P*=0.584) and OS (34.5 ± 12.3% vs 33.3 ± 19.2%; *P*=0.665).

### The Effect of SCT in Patients With *WT1* Mutations

**Figure 3:**
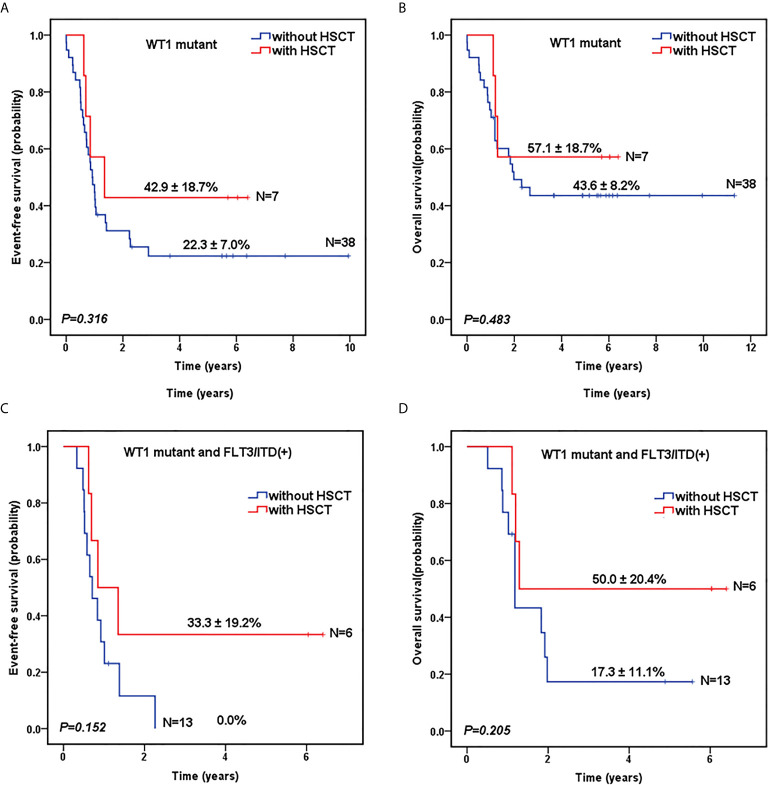
Survival curves of pediatric AML patients according to *WT1* mutations and hematopoietic stem cell transplantation (HSCT) status. Probability of EFS **(A)** and OS **(B)** for patients with *WT1* mutations according to HSCT status, respectively. Probability of EFS **(C)** and OS **(D)** for patients with *WT1* mutations and *FLT3*/ITD positive according to HSCT status, respectively.

As shown in **Table 1**, there was no significant difference in the proportion of HSCT in *WT1*-mutated group and *WT1* wild-type group (15.6% vs 16.2%, *P*=0.906). The survival analysis, after HSCT stratification, showed that for *WT1*-mutated pediatric AML patients, HSCT conferred a favorable prognostic impact with a trend of better 5-year EFS (42.9 ± 18.7% vs 22.3 ± 7.0% for chemotherapy-only; *P*=0.316) and OS (57.1 ± 18.7% vs 43.6 ± 8.2% for chemotherapy-only; *P*=0.483) (**Figures 3A, B**).

To further evaluate the role of HSCT in the patients with co-occurring *WT1* and *FLT3*/ITD mutations, we explored the impact of HSCT on those patients. As shown in **Figures 3C, D**, for AML patients with both *WT1* mutations and positive *FLT3*/ITD, 5-year EFS (33.3 ± 19.2%) and OS (50.0 ± 20.4%) were higher in children with HSCT than those with chemotherapy-only (EFS: 0.0 ± 0.0%, *P*=0.152; OS: 17.3 ± 11.1%, *P*=0.205), respectively, although the differences between the two groups were not statistically significant.

### Multivariate Analysis of Prognostic Factors

Cox regression analyses were then performed to evaluate *WT1* mutation status as a predictor of EFS and OS alongside other prognostic factors: age (utilizing 10 years of age as the cutoff value), white blood cell count at diagnosis (utilizing 50×10 ^9/^L as the cutoff value), high risk, standard risk, and HSCT. We identified *WT1* mutations as an independent prognostic factor for both EFS and OS in pediatric patients with AML (**Table 3**). *WT1* mutations were significantly associated with inferior EFS (HR: 1.910, 95% CI: 1.297-2.812, *P*=0.001) and OS (HR: 1.709, 95% CI: 1.090-2.679, *P*=0.020). Additionally, age (older than 10 years), white blood cell count greater than 50×10^9^/L at first diagnosis, high-risk and standard-risk were significantly related to poor EFS and OS, while HSCT was related to better survival prognosis (HR: 0.431, 95% CI: 0.313-0.593, *P*<0.001) and OS (HR: 0.594, 95% CI: 0.419-0.843, *P*=0.004).

**Table 3:** Cox regression analysis of *WT1* mutations and other prognostic factors.

Outcome	Variable	Hazard ratio (95% CI)	P-value
EFS	*WT1*	1.910(1.297-2.812)	0.001
	High risk	3.136(2.235-4.400)	<0.001
	Standard risk	2.581(2.207-3.286)	<0.001
	HSCT	0.431(0.313-0.593)	<0.001
	Age > 10 years	1.300(1.053-1.607)	0.015
	WBC>50×10^9^/L	1.499(1.220-1.841)	<0.001
OS	*WT1*	1.709(1.090-2.679)	0.02
	High risk	3.991(2.653-6.004)	<0.001
	Standard risk	3.413(2.494-4.670)	<0.001
	HSCT	0.594(0.419-0.843)	0.004
	Age > 10 years	1.496(1.158-1.933)	0.002
	WBC>50×10^9^/L	1.307(1.018-1.677)	0.036

CI, confidence interval; EFS, event-free survival; HSCT, hematopoietic stem cell transplantation; OS, overall survival; WBC, white blood cell count.

## Discussion

The TARGET program is a collaborative COG-national cancer institute (NCI) project aiming to comprehensively characterize the mutational, transcriptional, and epigenetic landscapes of a large, well-annotated cohort of pediatric cancer (20). Using this large cohort of subjects, we were able to investigate the clinical implication of *WT1* mutations in pediatric AML. Our findings showed that the frequency of *WT1* mutations was 6.7% among these 870 pediatric AML patients. This result was similar to the adult AML studies. In a large cohort of adult AML study, the frequency of *WT1* mutations among 3157 patients was reported to be 5.5% (21). Next, we found that *WT1* mutations were significantly associated with FAB subtypes of M4, with high white blood cell counts at first diagnosis, normal cytogenetics, and *FLT3*/ITD mutations. However, no association was found between *WT1* mutations and *CEBPA* mutations. These results were different from some of the other studies. For instance, a report by Ho et al. (14) also found that *WT1* mutations were related to normal cytogenetics and *FLT3*/ITD mutations, but they found no correlation between *WT1* mutations and white blood cell counts or M4 subtype. A pediatric AML report by Hollink et al. (13) showed that *WT1* mutations clustered significantly in the subgroup with normal cytogenetics and were associated with *FLT3*/ITD and *CEBPA* mutations.

The prognostic impact of *WT1* mutations has not been clarified in pediatric AML. In our study, we found that patients with *WT1* mutations had lower CR induction rates, worse EFS and OS rates in comparison to patients without *WT1* mutations. Patients with both *WT1* and *FLT3*/ITD mutations had a dismal prognosis. The multivariate analysis showed that *WT1* mutations were an independent adverse impact factor. These results are consistent with findings by Hollink et al. (13), though they found the CR induction rates did not differ significantly between patients with *WT1*-mutated and *WT1* wild-type AML. A report from the French study group confirmed that *WT1* mutations were an independent prognostic factor for pediatric AML (22). However, a report from the Japanese study group showed that *WT1* mutations were related to a poor prognosis in patients with normal cytogenetics, excluding those with *FLT3*/ITD and those younger than 3 years (23). By contrast, a report from the Nordic Society of Pediatric Hematology and Oncology (NOPHO) revealed that no significant correlation with survival was seen for *WT1* mutations (24). Notably, they found that patients with *WT1* mutations but negative *FLT3*/ITD had a superior EFS compared with patients with *WT1* wildtype with or without concurrent *FLT3*/ITD (24). In adult studies, the presence of *WT1* mutation has been found to be associated with poor clinical outcomes of AML patients in some but not all studies. In the studies from Cancer and Leukemia Group B (9) and Hou et al. (10), *WT1* mutations were correlated with a poor prognosis in AML patients. However, in the study from the German-Austrian Study Group (11), *WT1* mutation as a single molecular marker did not seem to impact the patient outcomes. These conflicting results may be due to the differences in sample size, exon of *WT1* mutations, and variable treatment protocols across studies. It has been reported that the negative impact of *WT1* mutations may be overcome by the use of repetitive cycles of high-dose cytarabine, especially in the subgroup of patients with negative *FLT3*/ITD genotype (11).

The mechanism of *WT1* mutations in leukemogenesis remains elusive. Several different *WT1* mutations have been described in AML, which occur primarily in exons 1, 7, and 9. *WT1* mutations may result in the loss of DNA binding ability due to loss of the zinc-finger domain or result in loss of expression of the *WT1* protein altogether (25–27). *WT1* mutations fail to properly direct the ten-eleven translocation-2 to its target sites, either by disruption of the interaction itself or by failing to bind to DNA (28, 29). Recently, Pronier et al. (30) have found that *WT1* heterozygous loss enhances stem cell self-renewal, *WT1* depletion cooperates with *FLT3*/ITD mutation to induce fully penetrant AML. Mutational analysis of a large cohort of AML cases revealed that *WT1* may play an important role in the epigenetic pathway (31, 32). Given the epigenetic alterations catalogued in *WT1* mutant, epigenetic-targeted therapy has been explored as a potential mechanism to deal with this subgroup of leukemia (33). Recently, Sinha et al. (34) have found that mutant *WT1* is associated with DNA hypermethylation of polycomb repressor complex 2 targets in AML, and inhibitor of enhancer of zeste homolog 2 (EZH2) may be helpful in this AML subtype.

Alternately, HSCT is one of the most effective treatments for AML. However, it is unknown whether *WT1-*mutated patients will benefit from HSCT. Our studies showed that compared to chemotherapy alone, HSCT tended to improve the prognoses of *WT1*-mutated patients, and for patients with both *WT1* and *FLT3*/ITD mutations as well. These results are in agreement with a previous pediatric AML report (14). Recently, Eisfeld et al. (12) have found that co-occurrence of *WT1* and *NPM1* mutations confers especially poor outcomes in a large cohort of 863 adult AML. They proposed that mutated *WT1* co-occurrence with mutated *NPM1* would be an adverse marker for risk stratification, indicating patients with both *WT1* and *NPM1* mutations might be considered for HSCT. However, since *NPM1* mutation is relatively rare in children, we could not draw a firm conclusion on this topic due to the small number of patients with both *WT1* and *NPM1* mutations. Thus, whether *WT1* mutation is an indication for HSCT in pediatric AML requires further investigation.

There were several limitations to our study. Firstly, since different *WT1* mutations may affect its functions on DNA binding or protein interaction differentially, the details of *WT1* mutants can be important to the clinical outcome of AML patients with these mutants. However, the information on the specific mutations of in *WT1* is not provided in the TARGET dataset, therefore, we can’t perform further analysis. Secondly, though this is a large pediatric AML cohort study, the sample size is still relatively small in the subgroups of patients with *WT1* mutations. We cannot rule out the contribution of *FLT3*/ITD co-occurrence towards the prognosis. Thirdly, our findings showed that *WT1* mutations were associated with poor clinical outcomes, and *WT1*-mutated patients might benefit from HSCT. These results suggested that *WT1* mutations could be used as predictive factors and linked to a specific clinical management plan. However, due to the limitations associated with the TARGET dataset as mentioned above, and the retrospective analysis nature of our study, a large multicentric prospective future study could be of value to further address the prognostic significance of *WT1* mutations in AML.

In summary, we analyzed the clinical implication of *WT1* mutations in a large pediatric AML cohort. Our findings showed that *WT1* mutations are independent poor prognostic factors in pediatric AML. Patients with co-occurring *WT1* and *FLT3*/ITD mutations had a dismal prognosis. Moreover, HSCT might be an effective strategy for patients with *WT1* mutations. These results have important implications and might contribute to the refining risk stratification of pediatric AML.

## Data Availability Statement

The original contributions presented in the study are included in the article/**Supplementary Material**, further inquiries can be directed to the corresponding authors.

## Ethics Statement

The studies involving human participants were reviewed and approved by TARGET Publications Committee. Written informed consent to participate in this study was provided by the participants’ legal guardian/next of kin.

## Author Contributions

LC and L-HX participated in project design, data collection, analysis, interpretation and manuscript drafting. YW participated in data interpretation and manuscript drafting. W-JW participated in data collection and analysis. D-HZ and J-PF participated in project design, data interpretation and manuscript drafting. SM participated in manuscript editing. All authors contributed to the article and approved the submitted version.

## Funding

This work was supported by the Natural Science Foundation of Guangdong Province, China (2018A030313680 to L-HX), Guangdong Basic and Applied Basic Research Foundation (2020A1515010312 to L-HX), Science and Technology Program of Guangzhou City, China (201803010032 to J-PF), Beijing Bethune Charitable Foundation (SCE111DS to J-PF), Xiu Research Fund (to LC).

## Conflict of Interest

The authors declare that the research was conducted in the absence of any commercial or financial relationships that could be construed as a potential conflict of interest.
