# Supplementary material for: Wilms Tumor 1 Mutations Are Independent Poor Prognostic Factors in Pediatric Acute Myeloid Leukemia
Source: Front Oncol. 2021 Apr 21;11:632094. doi: 10.3389/fonc.2021.632094 (PMC8096913; doi:10.3389/fonc.2021.632094)
Supplement: Supplementary file 1 [file DataSheet_1.pdf]

Supplementary Material

Supplementary Figures

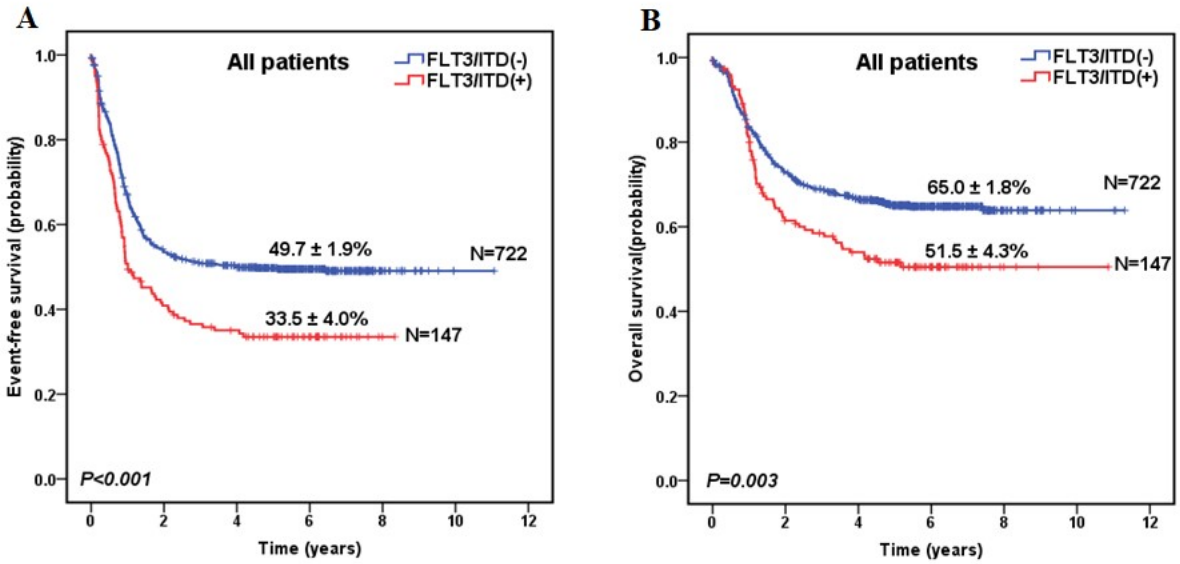

Supplementary Figure 1. Survival curves of pediatric AML patients according to the *FLT3*/ITD status. Probability of EFS (A) and OS (B) for patients according to *FLT3*/ITD status, respectively.

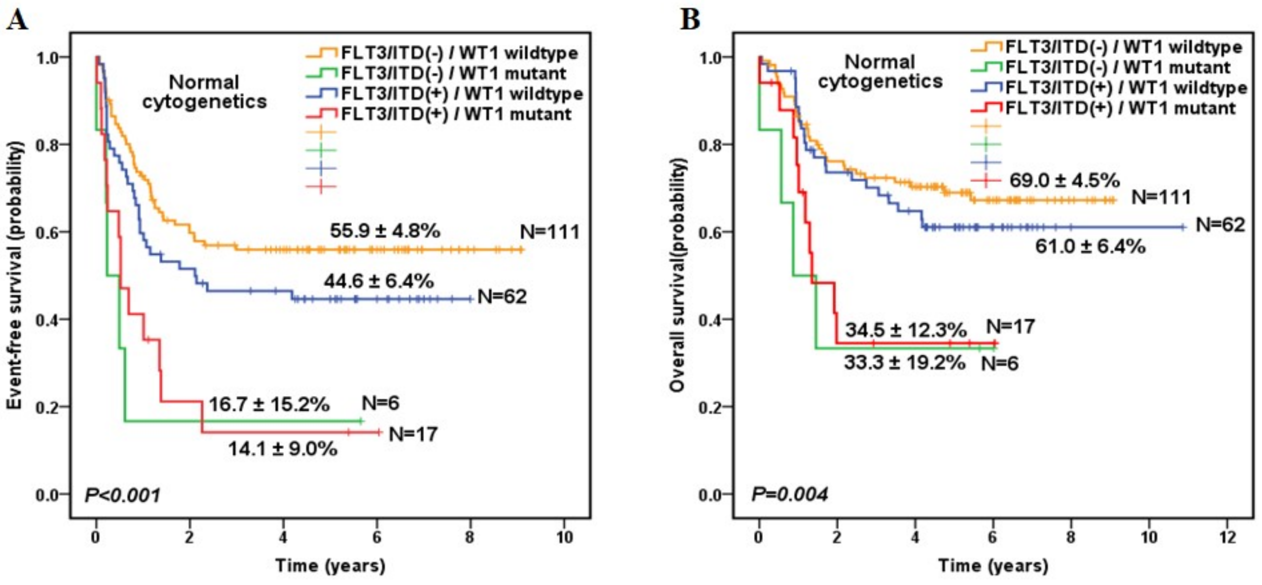

Supplementary Figure 2. Survival curves of the subgroup of cytogenetically normal AML patients according to the combined *WT1* and FLT3/ITD status. Probability of EFS (A) and OS (B) for patients according to the combined *WT1* and *FLT3*/ITD status, respectively.
